# Impact of post-procedural glycemic variability on cardiovascular morbidity and mortality after transcatheter aortic valve implantation: a post hoc cohort analysis

**DOI:** 10.1186/s12933-019-0831-3

**Published:** 2019-03-11

**Authors:** Guillaume Besch, Sebastien Pili-Floury, Caroline Morel, Martine Gilard, Guillaume Flicoteaux, Lucie Salomon du Mont, Andrea Perrotti, Nicolas Meneveau, Sidney Chocron, Francois Schiele, Herve Le Breton, Emmanuel Samain, Romain Chopard

**Affiliations:** 1 Department of Anesthesiology and Intensive Care, University Hospital of Besancon, 3 Boulevard Alexander Fleming, 25000 Besancon, France; 2 EA3920, University of Franche-Comte, 25000 Besancon, France; 3 Department of Cardiology, University Hospital of Brest, 29609 Brest, France; 4 Department of Vascular Surgery, University Hospital of Besancon, 25000 Besancon, France; 5 Department of Cardiothoracic Surgery, University Hospital of Besancon, 25000 Besancon, France; 6 Department of Cardiology, University Hospital of Besancon, 25000 Besancon, France; 7 Department of Cardiology and Vascular Diseases, University Hospital of Rennes, Rennes-1 University, LTSI, INSERM U1099, 35000 Rennes, France

**Keywords:** Aortic disease, Transcatheter aortic valve implantation, Blood glucose, Glucose variability, Adverse events

## Abstract

**Background:**

Glycemic variability is associated with worse outcomes after cardiac surgery, but the prognosis value of early glycemic variability after transcatheter aortic valve implantation is not known. This study was therefore designed to analyze the prognosis significance of post-procedural glycemic variability within 30 days after transcatheter aortic valve implantation.

**Methods:**

A post hoc analysis of patients from our center included in the FRANCE and FRANCE-2 registries was conducted. Post-procedural glycemic variability was assessed by calculating the mean daily δ blood glucose during the first 2 days after transcatheter aortic valve implantation. Major complications within 30 days were death, stroke, myocardial infarction, acute heart failure, and life-threatening cardiac arrhythmias.

**Results:**

We analyzed 160 patients (age (median [interquartile] = 84 [80–88] years; diabetes mellitus (n) = 41 (26%) patients; logistic Euroscore = 20 [12–32]). The median value of mean daily δ blood glucose was 4.3 mmol l^−1^. The rate of major complications within 30 days after procedure among patients with the lowest quartile of glycemic variability was 12%, increasing from 12 to 26%, and 39% in the second, third, and fourth quartiles, respectively. In multivariate analysis, glycemic variability was independently associated with an increased risk of major complications within 30 days after the procedure (odds ratio [95% CI] = 1.83 [1.19–2.83]; p = 0.006).

**Conclusions:**

This study showed that post-procedural glycemic variability was associated with an increased risk of major complications within 30 days after transcatheter aortic valve implantation.

*Trial registration* Clinical trial registration number https://www.clinicaltrials.gov/; identifier: NCT02726958; date: April 4th, 2016

## Background

Glycemic variability, defined as the degree of blood glucose level excursion over time, is associated with an increase in critically ill patients in-hospital mortality and poor prognosis after acute coronary syndrome [1–3]. In cardiac surgery patients, glycemic variability was reported to be associated with severe postoperative complications, regardless of the quality of blood glucose control obtained during the perioperative period [4, 5]. Glycemic variability was also reported to be a significant predictor of the length of intensive care unit (ICU) stay and acute kidney injury after cardiac surgery [6]. The mechanisms underlying the deleterious effect of glycemic variability include increased oxidative stress and endothelial dysfunction, and cell apoptosis [7, 8].

Transcatheter aortic valve implantation (TAVI) has emerged as a viable alternative to surgical aortic valve repair for patients with severe symptomatic aortic stenosis [9–11]. Although TAVI is not considered as a major surgical procedure, high post-procedure morbidity and mortality have been reported, mainly explained by the severe comorbidities presented by these patients [12, 13]. Although patient outcome after TAVI was not found to be different in diabetic and non-diabetic patients [14–16], Giannini et al. [17]. have recently suggested that post-procedural acute hyperglycemia increased the risk of acute kidney injury and was associated with higher mortality after TAVI. The link between post-procedural glycemic variability and outcome after TAVI is not known.

The purpose of this study was to investigate whether glycemic variability could be associated with an increased risk of major cardiovascular or cerebrovascular events or death within 30 days after TAVI.

## Methods

### Study design and setting

We conducted a *posthoc* analysis of a subgroup of patients included between February 2009 and June 2012 at our institution in the multicenter FRANCE or the FRANCE-2 registries [18, 19]. The registries were approved by the Institutional Review Board of the French Ministry of Health. All patients provided written informed consent to have their data included in the registries. The present study was conducted in accordance with the Code of Ethics of the World Medical Association forth in the Helsinki Declaration of 1975 and with French bioethics law (Art. L. 1121-1 of the law no. 2004-806, August 9th, 2004). It was approved by the Institutional Review Board of the French Society of Thoracic and Cardio-Vascular Surgery (no. CERC-SFCTCV-2016-2-17-17-18-2-BEGu, Chairperson Prof JL de Brux) on March 12th, 2016. The study was registered on April 4th, 2016 at http://www.clinicaltrials.gov (Identifier: NCT02726958). This manuscript adheres to the applicable Strengthening the Reporting of Observational studies in Epidemiology (STROBE) guidelines.

### Population of the study

Inclusion criteria for the FRANCE and FRANCE-2 registries were: (1) severe aortic stenosis, defined as an aortic valve area of < 0.8 cm^2^, a mean aortic-valve gradient ≥ 40 mmHg, or a peak aortic jet velocity ≥ 4.0 m s^−1^; (2) New York Heart Association (NYHA) class II, III, or IV symptoms; (3) non-eligibility for aortic surgery; and (4) aortic valve repair using TAVI. Eligibility of the patients for TAVI was assessed by a multidisciplinary team.

The choice of the TAVI device (self-expandable Medtronic CoreValve (Medtronic, Inc., Minneapolis, Minnesota, USA) or the balloon-expandable Edwards SAPIEN (Edwards Lifesciences, Irvine, California, USA), the route of implantation and the anesthetic protocol were left at the discretion of the physicians in charge of the patient. At the end of the procedure, all patients were admitted to the cardiac surgery ICU. Patients who suffered a major complication within the first 48 h after TAVI were excluded from the analysis.

Peri-procedural blood glucose management was performed by a dynamic insulin therapy protocol [20]. Briefly, the rate of insulin infusion was adjusted according to the current blood glucose value, the previous blood glucose value and the current rate of insulin infusion. All blood glucose values were obtained from glucose meter readings (Optium Xceed™, Abbott Diabetes. Care Ltd., Witney, UK) measured from an arterial blood sample taken from the indwelling arterial catheter. Blood glucose levels were checked hourly until 3 consecutive blood glucose values were within the target range, then every 3 h Hourly blood glucose measurement was resumed if any of the following occurred: change in the rate of insulin infusion, change in clinical condition, initiation or cessation of vasopressor therapy or renal replacement therapy. The intravenous insulin infusion was replaced by subcutaneous insulin when oral feeding was initiated. The insulin therapy protocol was managed by the nurses, and the medical team was consulted in case of severe hypoglycemia or uncontrolled hyperglycemia.

### Variables measurement

Baseline characteristics of patients and perioperative outcome data within 30 days after TAVI were extracted from the FRANCE and FRANCE-2 registries [18, 19]. For each patient, the number of blood glucose measurements performed, the average blood glucose value, the percentage of blood glucose value within the range of 4.4–8.2 mmol l^−1^, the number of adjustments made in the insulin infusion rate, and the rate of insulin therapy adjustment (defined as the ratio of the number of adjustment made in the insulin infusion rate on the number of blood glucose measurements performed) were recorded during the first 48 h after TAVI. All patients spent the first 48 h after the procedure in the intensive care unit (ICU), and then, were discharged to the ward in the absence of complication. To allow for a homogenous measurement of glycemic variability, the observation period of the study was defined as the first 48 h after TAVI. Glycemic variability was assessed using the following variables: (1) mean daily δ blood glucose, defined as the mean of the daily difference between the maximal and the minimal blood glucose value; [2] (2) standard deviation (SD) of blood glucose, defined as the SD of all blood glucose readings in a patient within the first 48 h after the procedure; and (3) the coefficient of variability of blood glucose level, expressed in percentage, defined as the ratio of the SD of blood glucose to the average of all blood glucose values * 100. The rate of moderate (blood glucose < 3.8 mmol l^−1^) or severe (< 2.2 mmol l^−1^) hypoglycemia [21], and the rate of hyperglycemia, defined as two consecutive blood glucose values ≥ 8.2 mmol l^−1^ were also recorded [17]. The insulin infusion rate variability was calculated from and expressed as the standard deviation of all insulin infusion rates per patient.

### Endpoints

The primary endpoint was the rate of post-procedural major adverse events (MAEs) occurring between the 3rd and the 30th day after TAVI. This composite endpoint included death from all causes, stroke, myocardial infarction, acute heart failure, and life-threatening ventricular arrhythmias, according to the Valve Academic Research Consortium criteria [22]. The primary endpoint was established before statistical analysis on the basis of the primary hypothesis of the study. Secondary endpoints were: (1) the rate of each event composing the primary outcome, occurring during the same period; (2) the rate of the following complications: pulmonary embolism, post-procedural atrial fibrillation, pericardial tamponade, respiratory failure requiring mechanical ventilation, and acute renal failure (graded according to the Acute Kidney Injury Network classification [23]); and (3) the length of stay in the Cardiac Surgery ICU.

### Statistical methods

The Shapiro–Wilk test was used to check whether quantitative data were normally distributed or not. Normally distributed data are presented as mean (SD), non-normally distributed data as median [interquartile range 25%–75%], and qualitative data as a number of patients (percentage).

Comparisons between patients with and without MAE were performed using the Student *t* test and the Mann–Whitney *U*-test for normally and non-normally distributed quantitative variables, respectively, and using the Chi square test for qualitative variables. The proportion of MAE in each quartile of the mean daily δ blood glucose was compared by using a Chi square test for trend. Stepwise logistic regression was performed to model the risk of post-procedural MAE. The logistic regression model included variables with a p-value of < 0.20 by univariate analysis comparing patients with and without post-procedural MAE (hypertension, obesity, New York Heart Association (NYHA) class III-IV, logistic Euroscore), hyperglycemia (yes/no) and quartiles of the mean daily δ blood glucose. The mean daily δ blood glucose was used to measure glycemic variability in the logistic regression model since this variable had the lowest p-value in the univariate analysis comparing patients with or without post-procedural MAE. This decision was made prior to statistical analysis considering the number of MAE observed. Discrimination and calibration of the model were tested using the Hosmer–Lemeshow test.

The relationships between the mean daily δ blood glucose and, respectively, the number of adjustments made in the insulin infusion rate, the rate of insulin therapy adjustment, and the insulin infusion rate variability, were analyzed by using the Spearman-rank correlation coefficient. The mean daily δ blood glucose was compared according to the preoperative glycosylated hemoglobin value ≥ 6.5% or < 6.5% [5] by using the Mann–Whitney *U*-test. No subgroup or sensitivity analysis were conducted.

All statistical analyses were performed using SAS 9.4 software (SAS Institute Inc., Cary, NC, USA). All p-values are two-sided and p < 0.05 was considered statistically significant. The post-study power was computed by using the Shieh-O’Brien approximation and estimated at 94%.

## Results

**Fig. 1:**
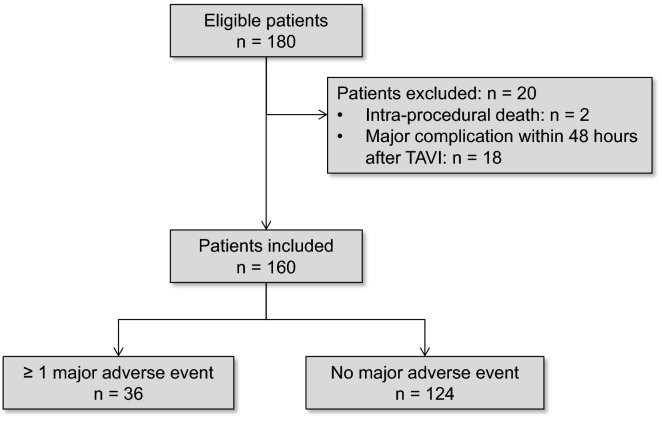
Flow chart of the study population

During the study period, a total of 180 patients were included in the FRANCE or FRANCE-2 registries in our center, and 160 patients were analyzed in the present study (Fig. 1). The demographic characteristics of the study population are detailed in Table 1. TAVI was performed via femoral route in 85 (53%) patients. A balloon-expendable valve was implanted in 148 (92%) patients.

**Table 1:** Baseline demographics and patient characteristics, in patients who underwent or not a post-procedural major adverse event

	All patients (n = 160)	MAE− (n = 124)	MAE+ (n = 36)	*p* value
Age (years)^a^	84 [80–88]	84 [81–88]	84 [79–87]	0.56
Male	79 (49)	60 (48)	19 (53)	0.64
Comorbidities				
Smoking	21 (13)	15 (12)	6 (17)	0.57
Dyslipidemia	77 (48)	61 (49)	16 (44)	0.62
Diabetes mellitus	41 (26)	32 (26)	9 (25)	1.00
Hypertension	119 (74)	89 (72)	30 (83)	0.16
Obesity (BMI ≥ 30 kg m^−2^)	25 (16)	23 (19)	2 (6)	0.07
COPD	119 (74)	94 (76)	25 (69)	0.44
Stroke	23 (14)	18 (15)	5 (14)	1.00
Peripheral artery disease	22 (14)	16 (13)	6 (17)	0.59
Coronary artery disease	100 (62)	79 (64)	21 (58)	0.56
Medications				
Beta-blockers	27 (17)	22 (18)	5 (14)	0.62
ECA	55 (34)	42 (34)	13 (36)	0.94
Statin	69 (43)	55 (44)	14 (39)	0.39
Aspirin	71 (44)	55 (44)	16 (44)	0.82
Clopidogrel	47 (29)	37 (30)	10 (28)	0.83
Biguanides	9 (6)	9 (7)	0 (0)	0.12
Insulin	8 (5)	6 (5)	2 (6)	1.00
Previous CABG	38 (24)	30 (24)	8 (22)	1.00
NYHA class III or IV	95 (59)	69 (56)	26 (72)	0.07
Pacemaker	23 (14)	16 (13)	7 (19)	0.42
Atrial fibrillation	38 (24)	31 (25)	7 (19)	0.66
Logistic Euroscore (%)^a^	20 [12–32]	20 [12–30]	26 [14–36]	0.09
Baseline eGFR (ml min^−1^ 1.72 m^−2^)^a^	42 [30–58]	42 [29–60]	40 [30–46]	0.21
Preoperative blood glucose level (mmol l^−1^)^a^	5.3 [4.7–6.1]	5.3 [4.6–6.1]	5.2 [4.9–5.7]	0.83
Glycosylated hemoglobin (%)^a^	6.0 [5.5–6.8]	6.0 [5.5–6.8]	5.8 [5.5–6.5]	0.55
Left ventricular function				0.60
Good (LVEF ≥ 50%)	108 (68)	86 (70)	22 (61)	
Fair (LVEF 30–49%)	47 (29)	34 (27)	13 (36)	
Poor (LVEF < 30%)	5 (3)	4 (3)	1 (3)	
Mean transaortic gradient (mm Hg)^a^	49 [40–57]	49 [42–57]	46 [35–57]	0.40

Data are number of patients (percentage)

BMI, body mass index; CABG, coronary artery bypass graft; COPD, chronic obstructive pulmonary disease; ECA, enzyme conversion antagonist; eGFR, estimated glomerular filtration rate; LVEF, left ventricular ejection fraction; MAE, major adverse event; NYHA, New York Heart Association; STS, Society of Thoracic Surgery

^a^Data are median [interquartile range 25–75%]

The primary endpoint occurred in 36 (22%) patients. Nine (6%) patients had 2 or more post-procedural MAEs. Baseline characteristics did not significantly differ between those who suffered an MAE and those who did not (Table 1). The rates of each event composing the primary outcome and of other complications (secondary outcomes) are given in Table 2. The length of stay in the Cardiac Surgery ICU was significantly higher in patients with post-procedural MAE versus those without (4 [2–6] vs. 2 [1–3] days, p < 0.001).

**Table 2:** Rate of post-procedural adverse events

	All patients (n = 160)
Major adverse event	36 (22)
Death	17 (11)
Myocardial infarction	6 (4)
Stroke	7 (4)
Acute heart failure	12 (7)
Life-threatening cardiac arrhythmia	6 (4)
Other postoperative complication	60 (37)
Atrial fibrillation	45 (28)
Respiratory failure requiring mechanical ventilation	13 (8)
Acute renal failure	50 (31)
AKIN grade 1	31 (19)
AKIN grade 2	4 (2)
AKIN grade 3	15 (9)

Data are number of patients (percentage)

Major adverse event is a composite endpoint including death from all causes, stroke, myocardial infarction, acute heart failure, and life-threatening ventricular arrhythmias

AKIN, Acute Kidney Injury Network classification, used for acute renal failure grading

Variables related to blood glucose control within the first 48 h after TAVI are presented in Table 3. The average blood glucose level, the percentage of blood glucose values within the target range of 4.4–8.2 mmol l^−1^ and the rate of hyperglycemia or severe hypoglycemia were not different between patients who underwent post-procedural MAE and those who did not (Table 3).

**Table 3:** Comparison of blood glucose control parameters within the first 48 after TAVI between patients who underwent or not a post-procedural major adverse event

	MAE− (n = 124)	MAE+ (n = 36)	*p* value
Blood glucose control parameters			
Number of blood glucose measurements per patient^a^	16 [12–19]	18 [14–20]	0.03
Average blood glucose value (mmol l^−1^)^a^	7.1 [6.6–7.8]	7.2 [6.5–8.1]	0.54
Percentage of blood glucose values within 4.4–8.2 mmol l^−1^	67 [51–79]	58 [41–75]	0.08
Hyperglycemia^b^	77 (62)	26 (72)	0.26
Moderate hypoglycemia^c^	20 (16)	9 (25)	0.23
Severe hypoglycemia^d^	3 (2)	2 (6)	0.31
Insulin consumption			
Total dose of insulin infused (IU/l)^a^	12 [4–19]	13 [3–30]	0.40
Glycemic variability			
Mean daily δ blood glucose (mmol l^−1^)^a^	4.0 [2.9–5.3]	5.4 [4.1–7.7]	0.001
Standard deviation of blood glucose value (mmol l^−1^)^a^	1.6 [1.1–2.0]	1.8 [1.5–2.4]	0.01
Coefficient of variability of blood glucose (%)^a^	22 [17–27]	24 [22–34]	0.007

Data are number of patients (percentage)

MAE, major adverse event

^a^Data are median [interquartile range 25–75%]

^b^Hyperglycemia was defined as two consecutive blood glucose values ≥ 8.2 mmol l^−1^

^c^Moderate hypoglycemia was defined as a blood glucose value < 3.8 mmol l^−1^

^d^Severe hypoglycemia was defined as a blood glucose value < 2.2 mmol l^−1^

The glycemic variability, as assessed using mean daily δ blood glucose, SD of blood glucose or coefficient of variability, was significantly higher in patients with post-procedural MAE (Table 3). The median value of mean daily δ blood glucose was 4.3 mmol l^−1^, and the 1st, 2nd, 3rd and 4th quartile of the mean daily δ blood glucose values were < 3.0, 3.0–4.3, 4.4–5.7, > 5.7 mmol l^−1^, respectively. The proportion of MAE within 30 days after TAVI significantly increased for each quartile of the mean daily δ blood glucose, from 12% and 12% in the 1st and 2nd quartiles to 26%, and 39% in the third, and fourth quartiles, respectively (p = 0.002). The mean daily δ blood glucose did not significantly differ in patients presenting post-procedural atrial fibrillation (4.2 [3.0–5.7] vs 4.3 [2.9–5.8] mmol l^−1^, p = 0.93) and in patients suffering from acute renal failure (4.1 [2.5–5.8] vs 4.4 [3.2–5.7] mmol l^−1^, p = 0.34) within 30 days after TAVI.

By logistic regression, mean daily δ blood glucose was associated with an increased risk of post-procedural MAE within 30 days after TAVI (odds ratio [95% confidence interval] per quartile: 1.83 [1.19–2.83]; p = 0.006) (Table 4).

**Table 4:** Multivariable analysis of factors associated with an increased risk for major adverse event within 30 days after TAVI

	OR [95% CI]	*p* value
Mean daily δ blood glucose, per quartile^a^	1.83 [1.19–2.83]	0.006
Hyperglycemia^b^	0.84 [0.31–2.27]	0.73
Logistic EuroSCORE (per % of increase)	1.03 [0.99–1.06]	0.12
NYHA class III or IV	2.00 [0.83–4.81]	0.12
Obesity (BMI ≥ 30 kg m^−2^)	0.22 [0.05–1.05]	0.06
Hypertension	1.64 [0.57–4.72]	0.36

Chi2 = 6.13, p = 0.63 for the Hosmer–Lemeshow goodness-of-fit test

BMI, body mass index; NYHA, New York Heart Association; OR [95% CI], odds ratio [95% confidence interval]

^a^Study population divided into 4 equal groups according to the quartile of mean daily δ blood glucose defined by values < 3.0, 3.0–4.3, 4.4–5.7, > 5.7 mmol l^−1^, respectively

^b^Post-procedural hyperglycemia was defined as two consecutive blood glucose values ≥ 8.2 mmol l^−1^

**Fig. 2:**
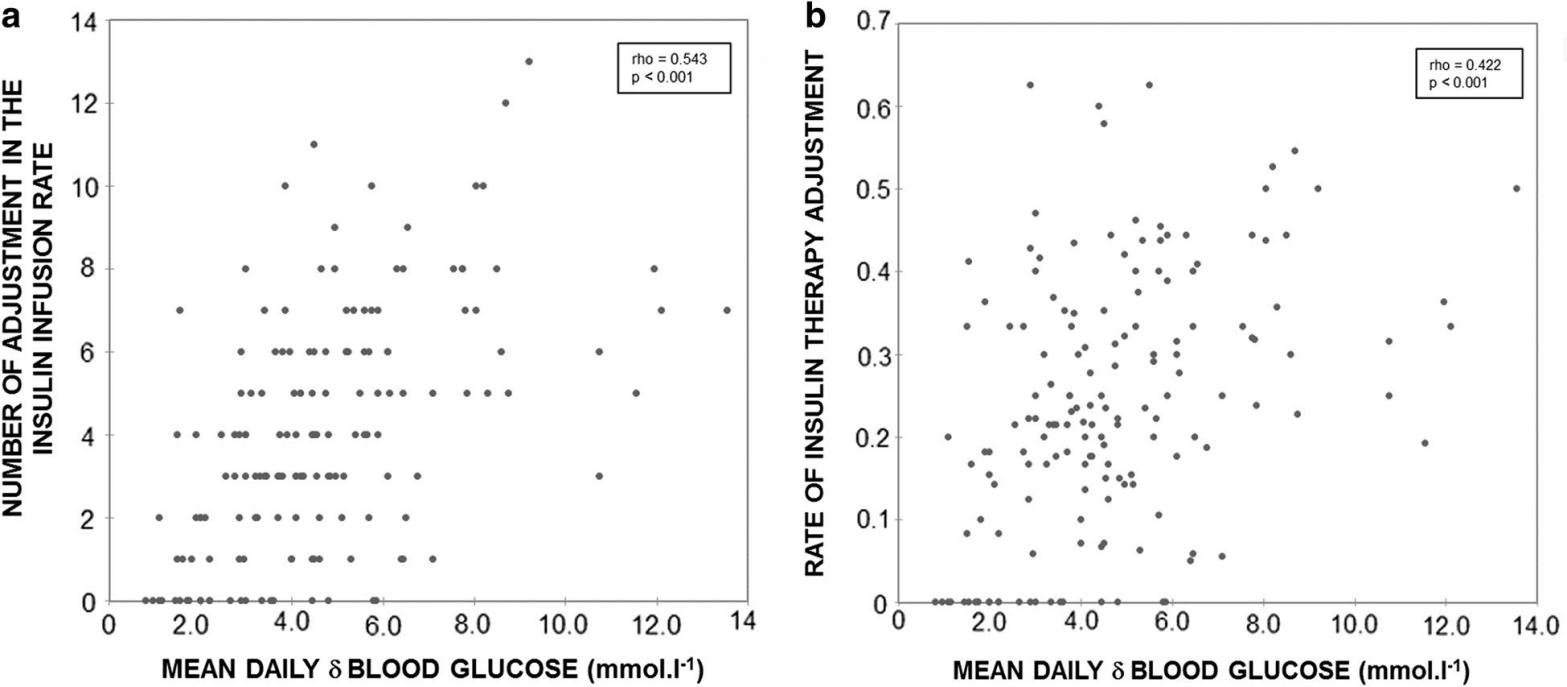
Glycemic variability and insulin therapy adjustments. **a** Relationship between mean daily δ blood glucose and the number of adjustments made in the insulin infusion rate. **b** Relationship between mean daily δ blood glucose and the rate of insulin therapy adjustments

The median number of adjustments made in the insulin infusion rate was 4 [2–6], and the median rate of insulin therapy adjustment was 0.22 [0.14–0.33]. A statistically significant correlation was found between the mean daily δ blood glucose and (1) the number of adjustments made in the insulin infusion rate (Spearman δ = 0.543; p < 0.001); (2) the rate of insulin therapy adjustment (Spearman δ = 0.422; p < 0.001); and (3) the insulin infusion rate variability (Spearman δ = 0.564; p < 0.001) (Fig. 2a, b). The mean daily δ blood glucose did not significantly differ according to the preoperative glycosylated hemoglobin (5.4 [3.2–7.1] versus 4.2 [2.9–5.6] mmol l^−1^, p = 0.06; in patients with preoperative glycosylated hemoglobin ≥ and < 6.5% respectively).

## Discussion

Our results suggest that early post-procedural glycemic variability was associated with a higher risk for MAEs within 30 days after TAVI. In contrast, the average blood glucose value and the rate of hyperglycemic episodes within the first 2 days post-procedure were not found to be related to outcome.

### Glycemic variability and outcome

Long-term visit-to-visit glycemic variability was associated to both a higher risk of micro- and macrovascular complications and poor outcome after acute lung diseases in type 2 diabetes mellitus patients [24–26]. The detrimental impact of short-term glycemic variability on the outcome of both diabetic and non-diabetic patients has been reported in various clinical situations [1, 2, 27, 28]. In cardiac surgery patients, a strong association between glycemic variability and postoperative morbidity and mortality was reported after CABG but not after isolated aortic valve surgical repair [5, 6, 29]. This suggests that several differences may exist between surgical aortic valve repair and TAVI, and deserves further analysis. First, TAVI patients included in the FRANCE and FRANCE-2 registries in our center were older (84 [80–88] versus 67 [57–76] years), and had more associated co morbidities and a higher rate of MAEs than patients who underwent cardiac surgery in the study by Bardia et al. [18, 19, 29]. This is also in accordance with previously published data evaluating patient outcome after TAVI [12, 18, 30]. Second, less blood glucose level variations was reported in the study by Bardia et al. than in ours [29]. This may be related to differences in patient case-mix or in the way blood glucose was managed postoperatively. It should be outlined that we observed a lower rate of MAEs among patients in the 2 first quartiles of glycemic variability, and increasing rates across the third and fourth quartiles, up to 44%. This is in accordance with the results of Krinsley et al. showing a link between the magnitude of blood glucose variability and outcome in ICU patients [27]. Lastly, recordings of blood glucose levels were limited to the first postoperative day in the study by Bardia et al., and evolution of glycemic variability on postoperative day 2 was not known [29].

### Parameters used to assess short-term glycemic variability

Several variables have been proposed to characterize glycemic variability, but no gold standard has emerged from previous studies. The hyperglycemic index [31], the hypoglycemic index, the glycemic penalty index [32], or the jack-knife approximate entropy [2] are well-adapted to quantify glycemic variability over a long period in diabetic patients. We used three variables, namely the standard deviation of blood glucose value [31], the coefficient of variability of blood glucose [32], and the mean daily δ blood glucose [2], all previously described to assess glycemic variability after surgery or in ICU patients. All three variables were significantly higher in patients who presented an MAE after TAVI. We chose to include the mean daily δ blood glucose in the logistic regression model since the p-value for this parameter in the univariate analysis was the lowest among the different glycemic variables examined. Moreover, the mean daily δ blood glucose could appear as the simplest variable to appreciate glycemic variability at the bedside in the absence of continuous glucose monitoring system.

### Underlying mechanisms for increased glycemic variability

Several factors have been reported to increased blood glucose variability, such as poorly-controlled diabetes mellitus or the degree of activation of the autonomic nervous system [33, 34]. We observed a significant but weak correlation between the mean daily δ blood glucose and both the number of insulin rate adjustments and the insulin infusion rate variability. These differences in blood glucose management could be a part of the mechanisms involved in, but also simply the consequences of increased glycemic variability.

### Limits of the study

The present study has several limitations. First, it was a single center study with relatively small sample size that could limit the external validity of the results observed. However, the baseline characteristics of the patients, the rate of complications and the mortality observed in this cohort were similar to the data reported in the entire cohort of the FRANCE and FRANCE-2 registries [18, 19]. Moreover, the values of the parameters used to assess glycemic variability observed in our cohort were similar to those reported in previously published studies [2, 6]. The sample size did not allow for subgroup analysis to differentiate outcome in either diabetic or non-diabetic patients and this question should be addressed in a specifically designed study. The sample size could also explain that the trend observed between the risk of MAEs and the Euroscore value did not reach the statistical significance although a weak but statistically significant association was reported between Euroscore and mortality after TAVI in some previously published studies [35, 36]. Second, glycemic variability was not determined using a continuous monitoring system and could be underestimated [3]. The present study is based on a *posthoc* analysis of data from patients included in the FRANCE and FRANCE-2 registries and continuous glucose monitoring is not our current practice in TAVI patients. Third, we used a composite parameter as the primary outcome to increase the power of the study. The choice of the events defining the primary outcome was based on their common potential impact on the prognosis of these patients. Fourth, we calculated a high post-study power, but potential unidentified confounding factor could not be excluded. Moreover, the power of the study could also be appropriately appreciated by analyzing the width and the magnitude of the 95% confidence intervals. Fifth, glycemic variability after discharge from the ICU was not measured and could have impacted differently the prognosis of these patients.

## Conclusions

Post-procedural glycemic variability could be associated with an increased risk of major adverse events within 30 days after TAVI. This observation needs to be confirmed in a specifically designed study involving a continuous glucose monitoring system. Glycemic variability could be a new dimension in the management of blood glucose after TAVI. Further investigations are warranted to identify the underlying mechanisms that contribute to increased glycemic variability and could represent a new therapeutic target to prevent wide variations in early post-procedure blood glucose levels.

## Abbreviations

AKIN: Acute Kidney Injury Network
BMI: body mass index
CABG: coronary artery bypass surgery
COPD: chronic obstructive pulmonary disease
eGFR: estimated glomerular filtration rate
ICU: intensive care unit
LVEF: left ventricular ejection fraction
MAE: major adverse event
NYHA: New York Heart Association
SD: standard deviation
STS: Society of Thoracic Surgery
TAVI: transcatheter aortic valve implantation
